# Deep sequencing and transcriptome analyses to identify genes involved in secoiridoid biosynthesis in the Tibetan medicinal plant *Swertia mussotii*

**DOI:** 10.1038/srep43108

**Published:** 2017-02-22

**Authors:** Yue Liu, Yi Wang, Fengxian Guo, Lin Zhan, Toni Mohr, Prisca Cheng, Naxin Huo, Ronghui Gu, Danning Pei, Jiaqing Sun, Li Tang, Chunlin Long, Luqi Huang, Yong Q. Gu

**Affiliations:** 1 College of Life and Environmental Sciences, Minzu University of China, Beijing 100081, China; 2 National Resource Center for Chinese Materia Medica, China Academy of Traditional Chinese Medicine, Beijing 100700, China; 3 USDA-ARS, Western Regional Research Center, Albany, CA 94710, USA; 4 Department of Plant Science, University of California Davis, Davis, CA 95616, USA

## Abstract

*Swertia mussotii* Franch. is an important traditional Tibetan medicinal plant with pharmacological properties effective in the treatment of various ailments including hepatitis. Secoiridoids are the major bioactive compounds in *S*. *mussotii*. To better understand the secoiridoid biosynthesis pathway, we generated transcriptome sequences from the root, leaf, stem, and flower tissues, and performed *de novo* sequence assembly, yielding 98,613 unique transcripts with an N50 of 1,085 bp. Putative functions could be assigned to 35,029 transcripts (35.52%) based on BLAST searches against annotation databases including GO and KEGG. The expression profiles of 39 candidate transcripts encoding the key enzymes for secoiridoid biosynthesis were examined in different *S*. *mussotii* tissues, validated by qRT-PCR, and compared with the homologous genes from *S*. *japonica*, a species in the same family, unveiling the gene expression, regulation, and conservation of the pathway. The examination of the accumulated levels of three bioactive compounds, sweroside, swertiamarin, and gentiopicroside, revealed their considerable variations in different tissues, with no significant correlation with the expression profiles of key genes in the pathway, suggesting complex biological behaviours in the coordination of metabolite biosynthesis and accumulation. The genomic dataset and analyses presented here lay the foundation for further research on this important medicinal plant.

The herbaceous genus *Swertia*, in the family Gentianaceae, contains traditional medicinal plants that have been used in China, India, Korea, and Japan for thousands of years. There are approximately 170 species of *Swertia*, with approximately 79 occurring in China^1^. Several species have pharmacological properties and are used in the treatment of different ailments^2^,^3^,^4^,^5^. For example, *Swertia chirata* is traditionally used as a bitter tonic to stimulate appetite and as a febrifuge in India, whereas *Swertia davidi* has been widely used as a remedy for acute bacillary dysentery. *Swertia mussotii* Franch is well known as “Di-da” and “Zang Yin Chen” in China because of its long history of use as a curative for hepatitis^6^,^7^ and has been recognized as one of the eight most important Tibetan traditional medicinal plants. *S*. *mussotii* has been widely used as a crude drug in more than 10 types of complex Tibetan folk medicine for the treatment of hepatitis, along with other uses that exploit its anti-diabetic and anti-hyperlipidaemic effects.

Chemical and pharmacological studies on *S*. *mussotii* have identified several major active compounds, swertiamarin, mangiferin, gentiopicroside, sweroside, oleanolic acid and three xanthones^8^,^9^,^10^,^11^,^12^. These compounds are of considerable interest as theraputic metabolites, and have been studied in wide-ranging pharmacological applications. For example, swertiamarin, a secoiridoid, had significant hepatoprotective effects in a liver damage model induced by alpha-naphthylisothiocyanate^13^. Recent research has reported that swertiamarin attenuates liver injury, inflammation, and cholestasis in bile duct-ligated rats^14^. Swertiamarin can also significantly up-regulate the hepatic bile acid detoxification enzymes and efflux transporters in rats, resulting in an increase in the water solubility of hydrophobic bile acids and the elimination of conjugated bile acids^15^.

The natural population of *S*. *mussotii* is restricted to high altitudes (2,000 to 5,000 metres above sea level) in the Qinghai-Tibetan Plateau area in China. The natural resource of *S*. *mussotii* yields less than 50 tons every year, while the high demand of the crude drug market requires several times more than this yield^1^. With the increasing demand for the species for medicinal use and the harvesting of flowering plants before fruit maturation^1^, wild populations of *S*. *mussotii* are being exhausted because of over-collection^16^,^17^. *S*. *mussotii* has been listed as an endangered species by some local governments in China, and harvesting wild plants is now prohibited in some areas of its natural distribution^18^. Domestic cultivation of *S*. *mussotii* could potentially solve the problem of high demand, but its seeds have strong dormancy and very low germination efficiency. In addition, there are concerns regarding the possible changes in the bioactive compound contents through domestic cultivation. When seven pharmacologically active compounds were compared between plants from natural, high-altitude populations and cultivated plants at low altitudes, mangiferin, one of the most abundant and active compounds was significantly lower in the cultivated sample, although most of the compounds were not significantly different. If domestic cultivation of this medicinal crop is to be successful, we need a better understanding of the genetic underpinnings of the pathways producing these compounds.

The secoiridoid biosynthesis pathway is still largely unresolved in *S*. *mussotii*, although it is thought to be closely related to terpenoid backbone biosynthesis^19^,^20^. Only one full-length cDNA clone (*SmG10H*) encoding geraniol 10-hydroxylase has been isolated from *S*. *mussotii* using PCR with degenerate pairs of primers designed based on the *G10H* gene sequences from other species^21^. Geraniol 10-hydroxylase is thought to play an important role in iridoid monoterpenoid biosynthesis. Functional characterization by overexpressing *SmG10H* in transgenic *S*. *mussotii* calli increased the level of the *SmG10H* transcript and the swertiamarin content. In addition, treatment with methyl jasmonate (MeJA) also increased the transcription of this gene, with a concomitant increase in swertiamarin content^21^. Except for *SmG10H*, little is known about the functions and regulation of other genes associated with the secondary metabolic pathways in *Swertia mussotii*. Only eight *Swertia mussotii* sequences (*matK*, *rpl16*, *ITS*, *SmG10H*, and *ITS1-5*.*8S-ITS2*) have been deposited in the NCBI GenBank database^21^. Clearly, the lack of sequence information seriously impedes our understanding of the metabolic pathways through the molecular characterization of gene functions^22^. Therefore, a comprehensive research strategy is critical for improving our understanding the biology of this high-altitude plant for high yield and for elucidating the metabolic pathways leading to the biosynthesis of its active compounds for better pharmaceutical applications.

Transcriptomics using next-generation sequencing technology has proved to be a powerful and cost-effective approach that generates a large amount of transcribed sequence data useful for a wide range of research applications^23^,^24^. In this study, we established transcriptome databases from the root, stem, leaf, and flower tissues collected at the flowering stage of native-habitat-grown *S*. *mussotii*. Through comprehensive transcriptome data analyses, we identified thousands of genes and analysed a set of putative genes involved in the secoiridoid pathway. These transcriptome data represent the first genomic resource for *S*. *mussotii*, which not only lays the foundation for further functional genomics study, but will also aid in the identification, characterization, and genetic modification of secondary metabolic pathway genes in this important medicinal plant.

## Result and Discussion

### Illumina sequencing and read assembly

**Figure 1:**
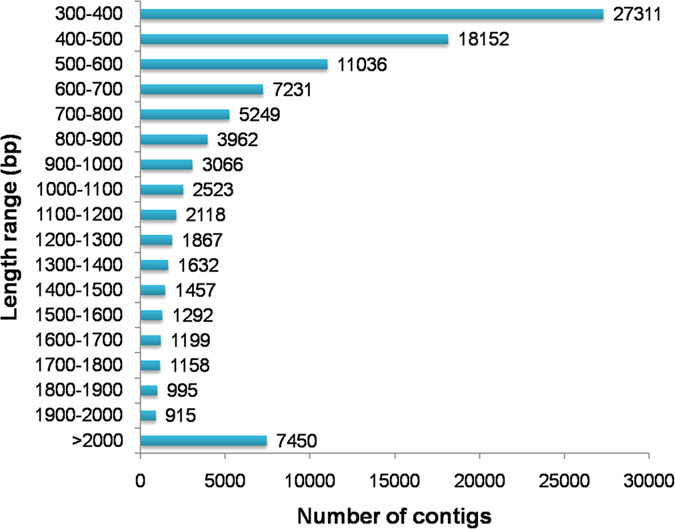
Overview of transcriptome assembly data showing the size distribution of transcripts.

*De novo* transcriptome analysis is an excellent platform for generating comprehensive information of the gene space in an organism for the discovery of novel genes, development of molecular markers, and construction of gene expression networks for specific tissues/organs. This approach is particularly useful for non-model species lacking whole-genome sequencing data. To generate a transcriptome database for *S*. *mussotii*, twelve RNAseq libraries were constructed from the root, stem, leaf, and flower tissues collected from three individual plants. A total of 1,295,381,780 raw pair-end reads with a read length of 100 bp were generated. After a trimming process was performed to remove adaptors, primer sequences, poly-A tails, and short and low quality sequences, 1,195,207,248 (92.27%) high-quality reads were recovered, ranging from 240 to 338 million for each tissue (Supplementary Table S1). The filtered reads were *de novo* assembled using CLC Genomics Workbench (v8.0.2). The CD-HIT tool was used to obtain non-redundant contigs. The final assembly dataset contained 98,613 unique contigs (transcripts) with an N50 of 1,085 bp. The unique contigs were designated *Swertia mussotii* tentative consensus (SmTc) transcripts and assigned unique identifier numbers from SmTc_1 to SmTc_98613. The total accumulated size of the assembled transcripts was approximately 82 Mb, with sizes ranging from 300–13,875 bp and an average size of 829 bp. There were 76,007 transcripts (77.08%) in the size range of 300–1,000 bp, 15,156 (15.37%) at 1,001–2,000 bp, and 7,450 (7.55%) >2,000 bp (Fig. 1).

### Functional annotation

Functional annotation of assembled transcripts provides insight into their molecular functions and biological processes in an organism. The *S*. *mussotii* transcripts were annotated based on protein similarity using BLAST searches against several public databases. The statistical summary for this annotation is shown in Supplementary Table S2. Among the 98,613 transcripts, 30,489 (30.92%) could be annotated in TrEMBL, 11,593 (11.76%) in Swiss-Prot, 11,175 (11.33%) in Clusters of Orthologous Groups (COG), 22,288 (22.60%) in Gene Ontology (GO), and 7,654 (7.76%) in the Kyoto Encyclopedia of Genes and Genomes (KEGG). Taken together, 35,029 transcripts (35.52%) could be assigned at least one putative function from one of these databases (Supplementary Table S2), while 64.48% of the transcripts had no significant protein matches (Supplementary Table S3). These transcripts may represent novel or diverse proteins and long non-coding RNAs in *S*. *mussotii*, or could be derived from less conserved 3′ or 5′ untranslated regions of the genes^25^,^26^.

**Figure 2:**
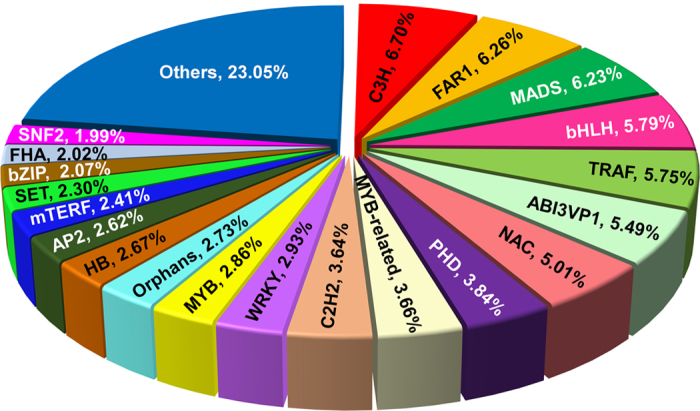
Distribution of transcription factor families.

Transcription factors (TFs) are diverse groups of gene families that play key regulatory roles in plant growth and development via controlling the expression of genes through specific binding to *cis*-regulatory elements present in the promoter regions of targeted genes^27^. Many TF families, including MYB, MYB-related, WRKY and bHLH, are known to regulate secondary metabolism biosynthesis in plants^28^,^29^,^30^. The number of genes encoding different TF families varies in different plant species and they often perform species-/tissue-specific or developmental stage-specific function(s)^31^. Our analysis of the *S*. *mussotii* transcriptome data revealed that 7,481 transcripts (7.59%) encode putative TFs that can be classified into 82 TF families (Supplementary Table S4). Members of the C3H transcription factor family were the most abundant (501, 6.70%) followed by FAR1 (468, 6.26%), MADS (466, 6.23%), bHLH (433, 5.79%) and TRAF (430, 5.75%) (Fig. 2). The identification of this large set of TFs, along with their expression profiling in individual tissues provides a rich resource for future characterization of specific TFs in various biochemical pathways in *S*. *mussotii*.

**Figure 3:**
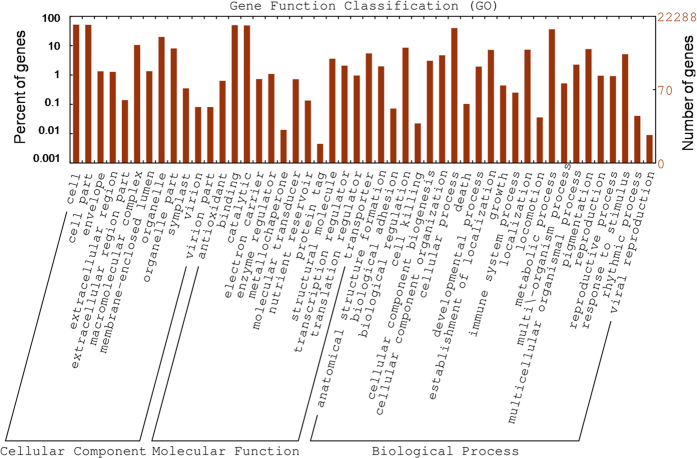
Frequencies and mean expression levels of transcripts matching GO terms. The percentage of transcripts matching GO terms is shown for each category as red bars.

When GO was used to classify gene functions, 22,288 transcripts could be assigned to one or more GO terms within the three domains and 22 functional categories (Fig. 3, Supplementary Table S5). Within the cellular component domain, the three most enriched categories were “cell” (11,559, 51.86%), “cell part” (11,422, 51.25%) and “organelle” (4,413, 19.80%). In the molecular function domain, the three most matched categories were “binding” (11,095, 49.78%), “catalytic activity” (10,891, 48.86%), and “transporter activity” (1,213, 5.44%). In the biological process domain, the three most common categories were “cellular process” (8,846, 39.69%), “metabolic process” (8,058, 36.15%), and “biological regulation” (1,904, 8.54%). The most commonly assigned functional categories in each domain were consistent with the results from other Gentianaceae species^32^,^33^.

**Figure 4:**
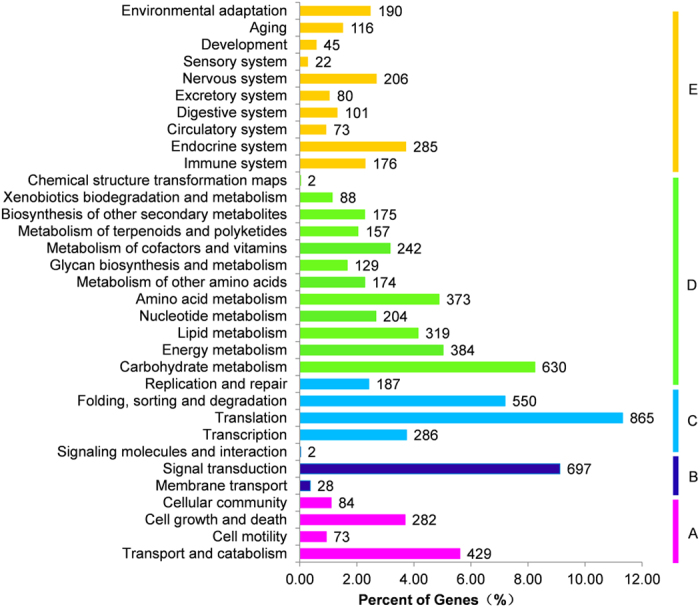
KEGG pathway classification map. Genes were divided into five branches according to the biological pathways they participated in: (**A)**, Cellular Processes; (**B**), Environmental Information Processing; (**C**), Genetic Information Processing; (**D**), Metabolism; (**E**), Organismal Systems.

The KEGG pathway database is a collection of maps representing the experimental knowledge concerning metabolism and provides various functions for genes in cells and organisms. We assigned gene functions to the biological pathways described in KEGG to better understand the biological functions of the genes in specific metabolic pathways (Fig. 4). To systematically analyse inner cell metabolic pathways and complex biological behaviours, we classified transcripts by mapping the annotated coding region sequences to the reference canonical pathways in the KEGG pathway database. From this analysis, we identified that 7,654 transcripts (7.76%) could be assigned to five main categories: “metabolism”, “genetic information processing”, “environmental information processing”, “cellular process”, and “organismal systems” with 33 sub-categories and 280 pathways (Fig. 4, Supplementary Table S6). The top 3 pathways with the largest numbers of mapped transcripts were “translations” (865 transcripts, 11.30%), “signal transduction” (697 transcripts, 9.11%), and “carbohydrate metabolism” (630 transcripts, 8.23%). Furthermore, we identified 643 protein-coding transcripts that have significant matches to 333 enzymes. These enzymes have assigned functions in 23 secondary metabolic pathways in KEGG (Table 1). Among them, 86 transcripts encode key enzymes involved in the pathways for terpenoid biosynthesis, including the synthesis of terpenoid backbone (54 transcripts), monoterpenoids (4 transcripts), diterpenoids (14 transcripts), and sesquiterpenoid and triterpenoid (14 transcripts). There were 123 transcripts found related to the flavonoid biosynthesis pathway including the phenylpropanoid (101 transcripts), flavonoid (18 transcripts), and flavone and flavonol biosynthesis pathways (4 transcripts). Only 35 transcripts were associated with alkaloid biosynthesis. Identification and future characterization of transcripts involved in different metabolic pathways will help us better understand their functions in the biosynthesis of active compounds in *Gentianaceae* plants.

### Simple sequence repeat (SSR) analysis

SSRs have been extensively used as molecular markers for various genotyping applications because they are abundant, easy to develop and detect, and highly polymorphic among species/accessions^34^,^35^. We screened the assembled transcript sequences using the MISA search tool and identified 33,529 SSRs (Supplementary Table S7). Among them, 8,778 SSRs (2–6 nt) were of the minimum length of 12 bp, with a frequency of one SSR per 9.3 kb of transcriptome sequences.

**Figure 5:**
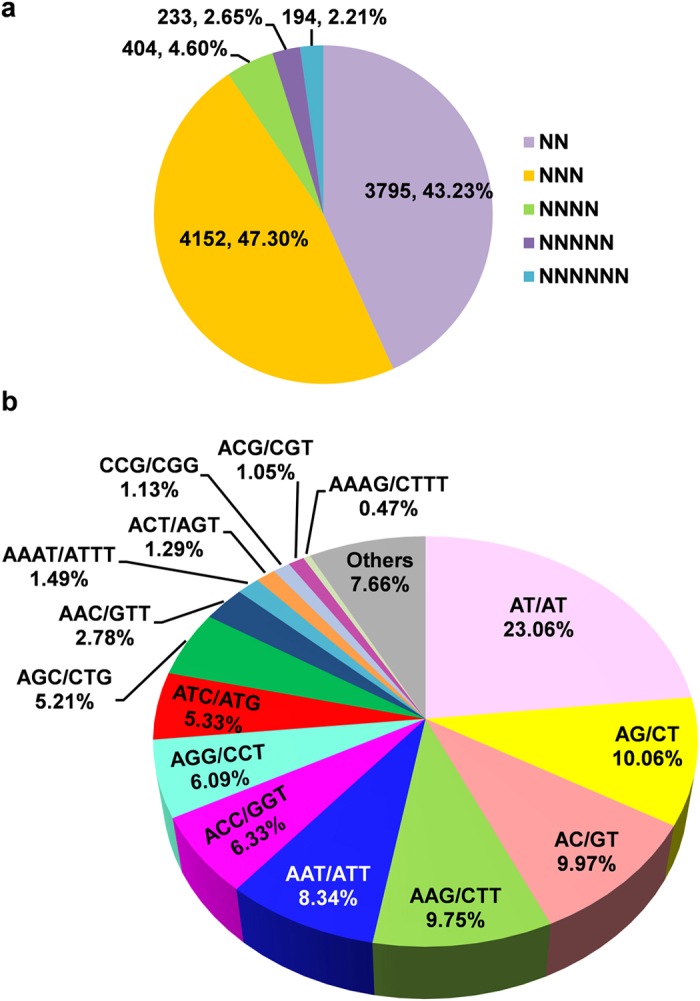
Simple sequence repeats (SSRs) in the *S*. *mussotii* transcriptome. (**a**) Distribution of different classes of SSRs. (**b**) Frequency of most abundant SSR motifs.

The identified SSRs were dominated by di and tri-nucleotide repeats representing approximately 43.23% (3,795) and 47.30% (4,152), respectively, of the SSRs with 2–6 nt (Fig. 5a). Among the di-nucleotide repeats, AT/AT was present at the highest (23.06%) frequency, followed by AG/CT (10.06%) and AC/GT (9.97%) (Fig. 5b). For tri-nucleotide repeats, AAG/CTT showed the highest occurrence (9.75%), followed by AAT/ATT (8.34%) and ACC/GGT (6.33%). AAC/GTT was the least abundant (2.78%) (Fig. 5b). This distribution is similar to those of other *Gentianaceae* family members, such as *Gentiana straminea*^33^ and *Veratrilla ballonii*^36^. Small fractions of tetra- (4.60%), penta- (2.65%) and hexa-nucleotide (2.21%) SSRs were also identified in *S*. *mussotii* (Fig. 5a). Given that the identified SSRs are present in transcripts, short repeat sequences could have played roles in gene evolution^37^,^38^. The availability of this large number of SSRs can greatly enhance large-scale genotyping studies for various applications in *S*. *mussotii*, such as genetic diversity assessment and association mapping for important traits.

### Differential gene expression analysis

**Figure 6:**
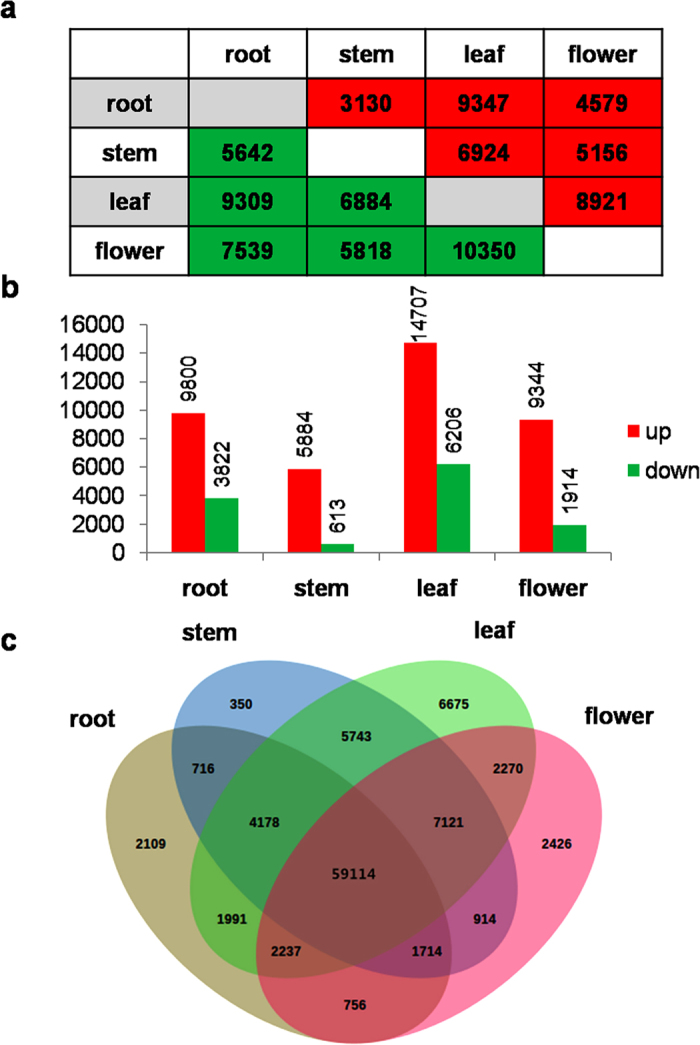
Differential expression analysis of *S*. *mussotii* transcripts. (**a**) Pair-wise comparisons of different tissues showing DETs. (**b**) The number of significantly (*P*-value ≤ 0.05 and at least two-fold change) up- and down- regulated transcripts in each tissue as compared to all three other tissues. (**c**) Venn diagram representing the number of DETs among *S*. *mussotii* tissues.

RNA-seq has emerged as a powerful technology to measure gene expression and tissue specificity at the whole-genome level^39^,^40^. To investigate the differential gene expression among different tissues, we mapped the high-quality reads from individual samples onto the *S*. *mussotii* transcriptome using CLC Genomics Workbench. Approximately 85–91% of the total reads were successfully mapped to the *S*. *mussotii* transcriptome (Supplementary Table S1). The differentially expressed transcripts (DETs) were identified based on the normalized RPKM (reads per kilobase of transcript per million mapped reads) value for each transcript in individual tissue (Supplementary Table S8) with Expander software (http://acgt.cs.tau.ac.il/expander/) using a t-test with a *p*-value cut-off ≤ 0.05 and at least a 2-fold expression change among different tissues^41^. Substantial transcriptional differences were seen in the pairwise comparisons between different tissues (Fig. 6a). The number of DETs was highest betweenthe flower and leaf and lowest between the stem and root (Fig. 6a). We further investigated DETs in one tissue in respect to the other three tissues. In this case, a DET was identified by testing its RPKM value from one tissue against the values from the three other tissues with the same statistical parameter. In the flowers, 9,344 transcripts were up-regulated and 1,914 transcripts were down-regulated relative to their expression in the three other tissues (Fig. 6b). Leaves had the largest number of up-regulated transcripts (14,707). In roots, 9,800 transcripts were up-regulated, while 3,822 transcripts were down-regulated. Stems had the smallest number of DETs, with 5,884 up-regulated and 613 down-regulated (Fig. 6b). In addition, we defined a transcript as having tissue-specific expression if its reads only came from a single tissue. A total of 11,560 transcripts exhibited tissue-specific expression, and 2,109, 350, 6,675 and 2,426 transcripts came from root, stem, leaf, and flower, respectively (Fig. 6c).

Among the identified DETs, 2,946 encoded transcription factors, and 565, 203, 1,046, and 1,132 came from the root, stem, leaf, and flower, respectively (Supplementary Table S4). Future characterization of specific transcription factors is required for a better understanding of the gene expression profiles and regulation in various biochemical pathways in *S*. *mussotii*. The DETs were further analysed using the KEGG database to explore their functional categories. Among 2,428 DETs with assigned functions in the biochemical pathways, 562, 114, 1,019, and 733 were from root, stem, leaf, and flower tissues, respectively, and these DETs were associated with 193, 60, 243, and 216 pathways in the corresponding tissues (Supplementary Table S9). These DETs had enrichment for 88, 23, 99, and 47 pathways in the root, stem, leaf, and flower tissues, respectively, while fifteen pathways were commonly enriched in all tissues, including the biosynthesis of secondary metabolites, microbial metabolism in diverse environments, starch and sucrose metabolism, amino sugar and nucleotide sugar metabolism in carbohydrate metabolism, the PI3K-Akt signalling pathway, the AMPK signalling pathway, and plant hormone signal transduction in the signal transduction group. We found that certain pathways were specifically enriched in particular tissues. The 25 pathways only enriched in the leaf were mainly related to energy metabolisms and signalling transductions. The 12 root enriched pathways were involved in the phosphonate and phosphinate metabolism pathways. In the flowers, specifically enriched pathways were primarily associated with secondary metabolism groups, including the flavonoid, sesquiterpenoid, and triterpenoid biosynthesis pathways. Pathways specifically enriched in the stem were not observed (Supplementary Table S9).

### Putative genes involved in the secoiridoids biosynthesis pathway

**Figure 7:**
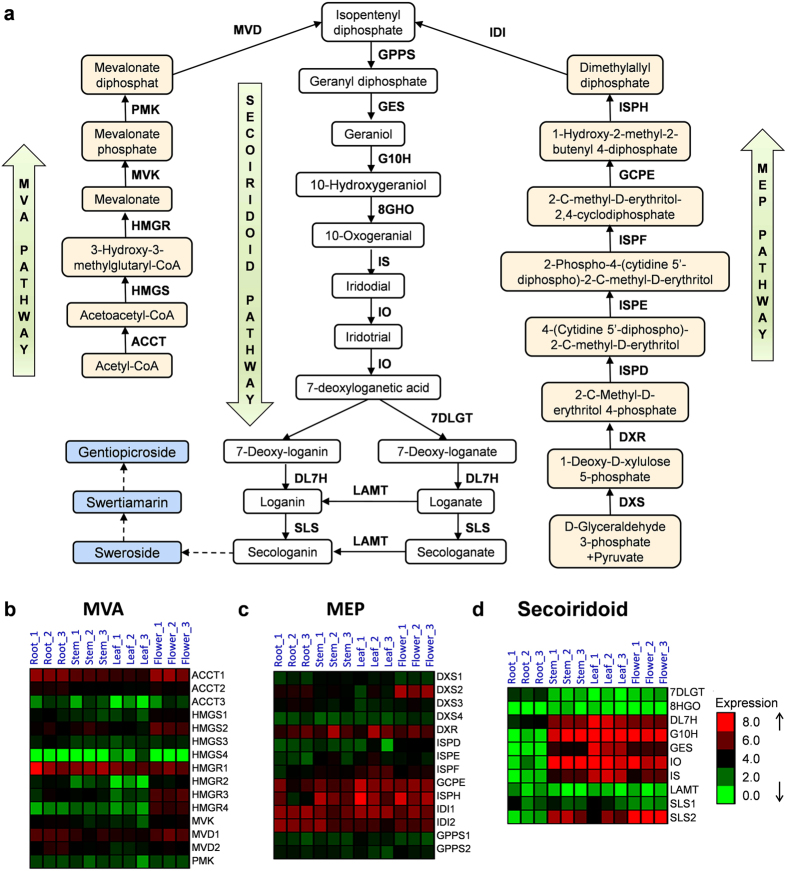
Schematic representation of the proposed swertiamatin biosynthesis pathway and the differential expression of genes involved in the pathway. (**a**) Proposed pathway of swertiamatin biosynthesis. (**b**) The expression of genes involved in the MVA pathway. (**c**) The expression of genes involved in the MEP pathway. (**d**) The expression of genes involved in the secoiridoid pathway.

In the proposed secoiridoid biosynthesis pathway (Fig. 7a), isopentenyl diphosphate (IPP) and its allylic isomer, dimethylallyldiphosphate (DMAPP), are the universal precursors in the first enzymatic step. These chemicals can be derived from both the cytosolic mevalonate (MVA) and/or plastidial methylerythritol phosphate (MEP) pathways. The prenyl-transfer of IPP on DMAPP catalysed by geranyl diphosphate synthase (GPPS)^42^ forms geranyl diphosphate (GPP), which is followed by the formation of the monoterpene geraniol by geraniol synthase (GES)^43^. It has been proposed that geraniol is the start of the secoiridoid pathway and that it is hydroxylated into 10-hydroxygeraniol by geraniol 10-hydroxylase^21^, leading to the formation of secologanin. Ultimately, secologanin is converted into swertiamarin, gentiopicroside, and other secoiridoid compounds through several currently unknown steps^44^. When examining the annotation of transcriptome contigs against the KEGG database, we identified 39 transcripts that could be classified into 24 enzyme categories associated with three metabolic pathways leading to secoiridoid biosynthesis (Table 2). It appeared that the genes encoding all of the enzymes in these pathways were identified in the transcriptome pool of *S*. *mussotii*. The analysis of the metabolic pathways with transcriptomic data help us identify the coding sequences of the genes involved in specific processes of the pathway in targeted plant species and cane aid in uncovering their family members and expression patterns in different tissues. For example, the reaction catalysed by the enzyme 3-hydroxy-3-methylglutaryl coenzyme A reductase (HMGR) is an important step in the MVA pathway^45^. The HMGR protein contains an N-terminal transmembrane domain, a highly divergent linker domain, and a C-terminal conserved catalytic domain^46^. The upregulation of *HMGR* genes could improve the yield of terpenes in transgenic plants^47^,^48^,^49^,^50^,^51^. The *HMGR* genes from a number of medicinal plants, including *Panax gingseng*, *Salvia miltiorrhiza*, *Panax notoginseng*, *Siraitia grosvenorii*, *Aquilaria sinensis*, and *Cyclocarya paliurus*, have been cloned and characterized. The *HMGR* gene family members range from 1 to 9 in different species, although most have fewer than three. In the Gentianaceae family, this gene has two copies in each species examined thus far, including *Gentiana rigescens*, *G*. *macrophylla* and *G*. *lutea*. In *S*. *mussotii*, we identified 4 full-length *HMGR* transcripts (*SmHMGR1*, *SmHMGR2*, *SmHMGR3*, *and SmHMGR4*) (Supplementary Figure S1). A sequence alignment revealed that these transcripts were conserved in the C-terminus region and diverged in the N-terminus region (Supplementary Figure S1). Transcriptome profiling data showed that *SmHMGR1* was constitutively highly expressed in all four tissues, while the other three genes all displayed differential expression patterns (Fig. 7b). Although the exact function of each *HMGR* gene is not clear in *S*. *mussotii*, we can speculate that *HMGR1* might play an important role in the pathway given its high expression level in all of the tissues.

Compared to the MVA and MEP pathways, the secoiridoid pathway is not been well understood. Recently, it has been shown that the *CrDXS* and *CrG10H* genes isolated from *Catharanthus roseus* are important genes in regulating terpenoid indole alkaloid biosynthesis^52^, filling important knowledge gaps in understanding the pathway. When we examined the expression profiles of the genes in the three pathways using the *S*. *mussotii* transcriptome data, it was noted that the transcripts of the MVA and MEP pathway genes, such as hydroxymethylglutaryl-CoA reductase (*SmHMGR*), diphosphomevalonate decarboxylase (*SmMVD*), hydroxymethylglutaryl-CoA synthase (*SmHMGS*), and 1-deoxy-D-xylulose-5-phosphate synthase (*SmDXS*), were more abundant in the flower tissues than in other tissues (Fig. 7b,c). However, in the secoiridoid pathway, five out of seven transcripts had high expression level in the stem, leaf and flower tissues but much lower expression in the root (Fig. 7c). Only the *Sm7DLGT* gene encoding 7-deoxyloganetic acid glucosyl transferase showed a higher expression in the root tissue than in the other three tissues (Fig. 7d). Two transcripts (*SmSLS1* and *SmSLS2*) both encode a secologanin synthase enzyme. The expression of the *SmSLS1* gene was constantly low in all of the tissues, while the expression of *SmSLS2* was higher in these tissues, particularity in the flower tissue (Fig. 7d).

Recently, a transcriptome study on a species (*Swertia japonica*) belonging to the same Swertia family was reported^53^. By comparing the transcriptome data from closely related species, we can identify transcripts that are shared and different in the two species and predict the evolutionary relationships of the key enzymes in the metabolic pathways. When the *S*. *mussotii* genes involved in the secoiridoid biosynthesis pathway were compared with those from *S*. *japonica*, we found that all of the genes identified in the pathway had good matches in the *S*. *japonica* transcriptome dataset, suggesting a conserved gene set in the pathway. The genes in the two species shared high sequence identity at the nucleotide level (Supplementary Table S10). Among these genes, only two had sequence identity between 80 to 85%; the rest (37) were over 90% identity. We further compared their expression patterns by examining the expression ratios of these pathway genes from leaf and root, the two tissues with transcriptome data available from both species (Supplementary Table S10). In the three pathways that are associated with the biosynthesis of secoiridoids, genes from the MVA pathway appeared to have similar ratios. The highest ratio difference was no more than four-fold between the two species. In both the secoiridoid and MEP pathways, genes with ratio differences more than 20-fold were noted, although many genes shared similar ratios. A better understanding of their differences in terms of gene expression and evolutionary relationship could be useful in the future comparison of metabolomics data to elucidate variations of the bioactive compounds in the two species.

### qRT-PCR validation of different expression patterns

**Figure 8:**
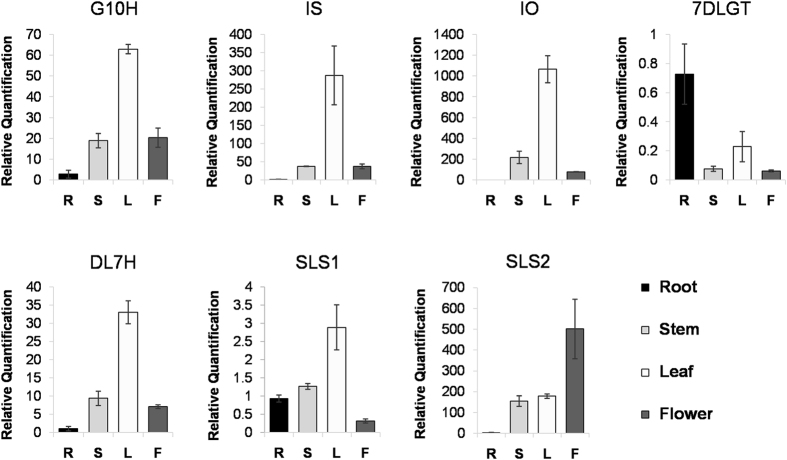
The expression pattern of seven genes in swertiamarin biosynthesis pathway across different tissues. R, root; S, stem; L, leaf; F, flower.

To validate the transcriptome analysis data and to provide a better understanding of the molecular basis of the metabolic pathways involved in swertiamarin biosynthesis, we selected 7 transcripts encoding key enzymes in the secoiridoid pathway to examine their expression patterns using qRT-PCR (Fig. 8). One of these transcripts, *SmG10H*, which encodes geraniol 10-hydroxylase, has been cloned in *S*. *mussotii* and has shown a potential role in swertiamarin biosynthesis^21^. Previous expression analysis of the *SmG10H* gene in leaf, stem, and root tissues indicated its highest expression level was in leaf tissue^21^. Our qRT-PCR data were consistent with this observation and showed that *SmG10H* expression in the flower was equivalent to that in the stem (Fig. 8). The higher expression of *G10H* in leaf tissue than root tissue has also been reported for *Gentiana rigescens* and *G*. *macrophylla*^32^,^54^. However, in *Swertia chirayita*, the *ScG10H* gene was most highly expressed in the roots^55^. Their different expression patterns could suggest that the regulation of *G10H* genes in the secoiridoid pathway might be different, even among closely related species.

Another well-characterized gene in the secoiridoid pathway is *7DLGT*, which encodes 7-deoxyloganetic acid glucosyl transferase. Previous reports have shown that the 7DLGT enzyme possesses a high catalytic efficiency towards 7-deoxyloganetic acid, and when its expression was reduced via virus-induced silencing in *C*. *roseus*, the secologanin levels declined significantly^56^. Secologanin is a key building block in the biosynthesis of many monoterpene indole alkaloids, including swertiamarin. Our qRT-PCR results showed that the expression pattern of *Sm7DLGT* was quite different from the other transcripts in the pathway. First, its expression level was low in all of the tissues compared to the expression levels of other transcripts in this pathway. Second, consistent with the transcriptome analysis data (Fig. 7c), *Sm7DLGT* was the only gene with its highest expression in the root tissue; similar to what was reported for *Swertia chirayita*^55^. The low expression of *Sm7DLGT* supports the hypothesis that it may be the rate-limiting enzyme in the secoiridoids biosynthesis pathway. Therefore, this gene could be a potential target for a biotechnological approach to engineer an improved the pathway for increasing swertiamarin yield in *S*. *mussotii* in the future.

Secologanin synthases (*SLS*) are P450 enzymes that catalyse an unusual ring-opening reaction using loganin in the biosynthesis of secologanin in the secoiridoid pathway. In *C*. *roseus*, there are two isoforms of *SLS*, *CrSLS1*(GenBank L10081) and *CrSLS2* (KF415117), with 94.0% nucleotide identity and the same enzymatic function^57^. However, the two *SLS* genes (*SmSLS1* and *SmSLS2*) from *S*. *mussotii* shared only 63.0% nucleotide identity, suggesting they have diverged extensively. While SmSLS2 (SmTc_16024) and CrSLS2 shared 74.4% amino acid identity, SmSLS1 (SmTc_4315) had the highest amino acid identity (49.8%) with the SLS protein from *Nothapodytes nimmoniana* based on the BLAST search result (Supplementary Figure S2)^58^. qRT-PCR indicated that the expression patterns of the two *SmSLS* genes were quite different. The highest expression of SLS1 was in the leaf and lowest in the flower, while the highest expression of SLS2 was in the flower and lowest in the root. Given the large differences at the sequence level and in their expression patterns, it is uncertain if SmSLS1 and SmSLS2 have similar biological functions in secologanin biosynthesis.

### Bioactive compound biosynthesis and accumulation

**Figure 9:**
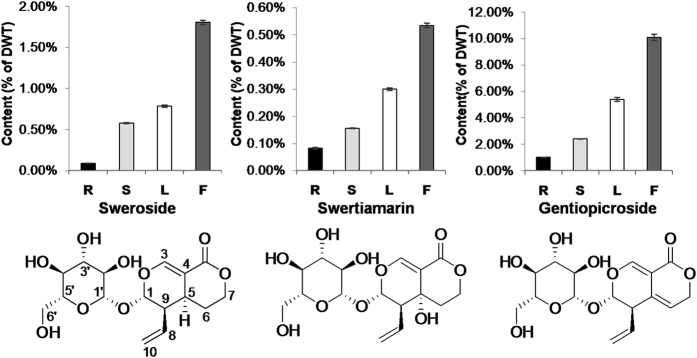
The content of sweroside, swertiamarin and gentiopicroside in different tissues. R, root; S, stem; L, leaf; F, flower.

To examine the possible associations of gene expression in the secoiridoid pathway with the metabolite products, we determined the contents of three bioactive compounds of the secoiridoid pathway, sweroside, swertiamarin, and gentiopicroside, in four tissues (root, leaf, stem, and flower). These compounds have similar chemical structures. Compared with gentiopicroside, on C-5, swertiamarin has a hydroxy group, while sweroside has a proton. Among the three compounds, gentiopicroside has the highest content in all of the tissues (Fig. 9). These compounds had similar accumulation patterns with the lowest content in the root and highest in the flower tissue. The high level of these metabolites in the flower tissue may have an ecological advantage for the plants, since they are bitter in taste. Thus, its high content may serve as a defence mechanism against predators. The low content in the root tissue showed a correlation with the relatively low expression of most biosynthesis genes except *7DLGT*. The highest content in the flower tissue did not correlate with the moderate relative expression of these genes. We also observed that the leaf tissue, which had the highest gene expression level, had a compound content level below that of the flower tissue. Taken together, the expression profiles of the metabolic biosynthesis genes in different tissues did not show significant correlations in relation to the metabolite contents. This result agrees with several previous reports, including the study in which the expression levels of the genes involved in the phenylpropanoid pathway did not show significant correlations with the accumulation pattern of shikonin^59^. An explanation for these observations could be that the highly expressed genes are involved in the biosynthesis of other secondary metabolites or that the biosynthesis is primarily controlled at the protein or enzyme levels. Alternatively, considering that the measured content reflects the accumulated level of the product, a transport mechanism wherein the metabolites are synthesized in the source (leaf) and rapidly transported into the sink (flower) might be involved.

In conclusion, we generated a comprehensive transcriptome assembly representing the gene space in *S*. *mussotii*. The deep transcriptome sequence data allowed us to identify and characterize the expressions levels of candidate transcripts encoding key enzymes involved in terpenoid metabolic pathways, providing insight into the biosynthesis of bioactive compounds in *S*. *mussotii*. The quantitative analyses of gene expression in relation to the bioactive compound contents across different tissues revealed the complexity of gene expression and metabolite accumulation in the secoiridoid biosynthesis pathway. Clearly, further corroboration through molecular, biochemical, enzymatic, and physiological studies will be needed to gain a better understanding of the underlying regulatory mechanisms involved. Nevertheless, the transcriptome data represent the first genomics resource for *S*. *mussotii* and lay the foundation for future research towards the improvement of this ethnomedicinal crop through genetics, genomics, and biotechnological approaches.

## Methods

### Plant material

Fresh roots, stems, leaves and flowers were collected from three individual *S*. *mussotii* plants at the full-bloom stage in their wild habitat located in Zhongda County in Yushu Tibetan Autonomous Prefecture of Qinghai Province. The samples were immediately frozen in liquid nitrogen and stored at −80 °C prior to RNA extraction.

### RNA isolation and transcriptome sequencing

Total RNA from the four tissues from three individual plants was extracted and processed separately as three replicates. The quality of the RNA samples was evaluated using a Bioanalyzer (Agilent Technologies, USA). RNAseq libraries were constructed with insert sizes of 200 to 500 bp, and were sequenced using the Illumina HiSeq 2000 platform with paired-end (PE) reads of 100 bp.

### *De novo* assembly and sequence processing

The raw data of the twelve RNA-seq reads were quality trimmed and then used for sequence assembly using CLC Genomics Workbench (v8.0.2) with the default parameters. For the removal of the redundant sequences, the CD-HIT^60^ package with an identity parameter of 95% was employed to generate a set of non-redundant contig sequence files. Contigs (transcripts) shorter than 300 bp were not included in the final *S*. *mussotii* transcriptome assembly dataset for further analyses.

### Gene function annotation

The consensus contig sequence files were annotated against the Nr (NCBI non-redundant protein sequences), PFAM (protein family), and Swiss-Prot (a manually annotated and reviewed protein sequence database) databases using local BLASTX program with an *E*-value threshold of 1e^−10^. GO analysis was performed using Blast2GO^61^ with the same E-value cutoff. To identify the transcription factors in the *S*. *mussotii* transcript dataset, we also applied the BLASTX search program against the plant transcription factor database^62^. Annotated sequences were further characterized based on KOG/COG classifications. Metabolic pathway mapping of the transcripts in the Nr Unigene set was performed using the KEGG Automatic Annotation Server^63^.

### Identification of SSRs

The transcripts sequences were scanned for the presence of SSRs using MISA (MIcroSAtellite) with default parameters^64^. The minimum number of repeat units for di-nucleotide was six, whereas for tri-, tetra-, penta- and hexa-nucleotide repeats, the minimum number of repeat units was greater than five in the search criteria.

### Differential expression analysis

To estimate the expression pattern of each transcript in different tissue samples, high-quality reads from each sample were mapped on the final transcriptome assembly using CLC Genomics Workbench (v8.0.2). A maximum of two mismatches was allowed for mapping. The read counts were normalized by calculating the RPKM for each transcript in an individual tissue. Differential gene expression analysis was performed using Expander (v7.1)^41^ software based on t-test. A *P*-value cut-off ≤0.05 (multiple tests correction: FDR) and a change of at least a two-fold change were used to identify significantly different transcript expressions levels.

### qRT-PCR analysis

Quantitative real-time PCR was performed using a QuantStudio Flex 6 system (Applied Biosystems, Foster City, CA, USA) with the Brilliant II SYBR Green QRT-PCR Master Mix Kit, 1-Step (Agilent Technologies, Cedar Creek, TX, USA). Gene-specific primers for the seven selected genes in the secoiridoid pathway were designed using Primer 3, and the primer sequences are listed in Supplementary Table S11. Real time PCR for each gene was performed using three biological replicates and three technical replicates. The tubulin transcript served as an internal control for normalization. The relative gene expression levels were calculated using the 2^−ΔΔCT^ method^65^.

### Extraction and estimations of sweroside, swertiamarin and gentiopicroside

Individual tissues (root, stem, leaf, and flower) were pooled from three plants. The dry materials were ground into fine powder. Approximately 50 mg of powder was extracted in 1 mL of methanol by ultrasonication at room temperature for 1 h, followed by centrifugation. The supernatant was then diluted to an appropriate concentration with methanol. Samples were separated using ultra high-performance liquid chromatography (UHPLC) with a HSS T3 column (2.1 × 100 mm, 1.7 μm, Waters) and a Thermo Scientific™ Dionex™ UltiMate™ 3000 Rapid Separation LC (RSLC) system. The mobile phase consisted of solvents (A), 0.10% aqueous formic acid, and (B), acetonitrile containing 0.10% formic acid. The gradient conditions were applied as follows: from 5.00% to 10.00% B in 1 min, from 10.00% to 60.00% B until 6 min, from 60.00% to 100.00% B in 6.5 min, maintained at 100.00% B for 3.5 min, from 10.1 min to 13 min, and maintained at 5.00% B. The flow rate was 300 μL/min, and the injection volume was 1 μL. Liquid chromatography-mass spectrometry (LC-MS) was performed using a Thermo Scientific™ Q Exactive™ Hybrid Quadrupole Orbitrap mass spectrometer equipped with a HESI-II probe and controlled with Xcalibur 2.2 SP1.48 software (Thermo Fisher Scientific)^66^. The sweroside, swertiamarin, and gentiopicroside content of each tissue was identified by the retention time and mass spectra data compared with standards, and their quantity was estimated by linear regression. The quantification was estimated in triplicate and the data were interpreted in terms of % of dry weight (DWT).

### Data availability

The raw Illumina data generated in this study were deposited in the NCBI Sequence Read Archive (SRA) under the BioProject accession number PRJNA320459.

## Additional Information

**Accession Codes**: The datasets of raw read sequences from each tissue were deposited in the NCBI Short Read Archive (SRA) database under the BioProject accession number PRJNA320459.

**How to cite this article:** Liu, Y. *et al*. Deep sequencing and transcriptome analyses to identify genes involved in secoiridoid biosynthesis in the Tibetan medicinal plant *Swertia mussotii*. *Sci. Rep.* **7**, 43108; doi: 10.1038/srep43108 (2017).

**Publisher's note:** Springer Nature remains neutral with regard to jurisdictional claims in published maps and institutional affiliations.

## Supplementary Material

Supplementary Figure S1,S2,S3, Table S1,S2,S10 and S11

Supplementary Dataset 1

Supplementary Dataset 2

Supplementary Dataset 3

Supplementary Dataset 4

Supplementary Dataset 5

Supplementary Dataset 6

Supplementary Dataset 7

## Figures and Tables

**Table 1:** Secondary metabolism pathways in *S*. *mussotii*.

Pathway ID	Pathways	Function categories	SmTc numbers
ko00100	Steroid biosynthesis	18	31
ko00130	Ubiquinone and other terpenoid-quinone biosynthesis	21	36
ko00230	Purine metabolism	91	160
ko00232	Caffeine metabolism	2	2
ko00400	Phenylalanine, tyrosine and tryptophan biosynthesis	23	41
ko00860	Porphyrin and chlorophyll metabolism	32	48
ko00900	Terpenoid backbone biosynthesis	28	54
ko00901	Indole alkaloid biosynthesis	1	1
ko00902	Monoterpenoid biosynthesis	2	4
ko00903	Limonene and pinene degradation	2	11
ko00904	Diterpenoid biosynthesis	8	14
ko00905	Brassinosteroid biosynthesis	7	7
ko00906	Carotenoid biosynthesis	21	30
ko00908	Zeatin biosynthesis	5	13
ko00909	Sesquiterpenoid and triterpenoid biosynthesis	8	14
ko00940	Phenylpropanoid biosynthesis	19	101
ko00941	Flavonoid biosynthesis	14	18
ko00942	Anthocyanin biosynthesis	3	5
ko00944	Flavone and flavonol biosynthesis	4	4
ko00945	Stilbenoid, diarylheptanoid and gingerol biosynthesis	5	14
ko00950	Isoquinoline alkaloid biosynthesis	9	15
ko00960	Tropane, piperidine and pyridine alkaloid biosynthesis	8	18
ko00965	Betalain biosynthesis	2	2

**Table 2:** Summary of transcripts in *S*. *mussotii* encoding enzymes involved in the secoiridoid pathway.

Pathway	Gene	Gene name	E.C. number	KO	SmTc
MVA	ACCT1	acetyl-CoA C-acetyltransferase	2.3.1.9	K00626	SmTc_2083
	ACCT2	acetyl-CoA C-acetyltransferase	2.3.1.9	K00626	SmTc_5029
	ACCT3	acetyl-CoA C-acetyltransferase	2.3.1.9	K00626	SmTc_33675
	HMGS1	hydroxymethylglutaryl-CoA synthase	2.3.3.10	K01641	SmTc_5929
	HMGS2	hydroxymethylglutaryl-CoA synthase	2.3.3.10	K01641	SmTc_7106
	HMGS3	hydroxymethylglutaryl-CoA synthase	2.3.3.10	K01641	SmTc_8664
	HMGS4	hydroxymethylglutaryl-CoA synthase	2.3.3.10	K01641	SmTc_81097
	HMGR1	hydroxymethylglutaryl-CoA reductase	1.1.1.34	K00021	SmTc_1585
	HMGR2	hydroxymethylglutaryl-CoA reductase	1.1.1.34	K00021	SmTc_10138
	HMGR3	hydroxymethylglutaryl-CoA reductase	1.1.1.34	K00021	SmTc_15910
	HMGR4	hydroxymethylglutaryl-CoA reductase	1.1.1.34	K00021	SmTc_22303
	MVK	mevalonate kinase	2.7.1.36	K00869	SmTc_7721
	PMK	phosphomevalonate kinase	2.7.4.2	K00938	SmTc_5121
	MVD1	diphosphomevalonate decarboxylase	4.1.1.33	K01597	SmTc_2684
	MVD2	diphosphomevalonate decarboxylase	4.1.1.33	K01597	SmTc_2834
MEP	DXS1	1-deoxy-D-xylulose-5-phosphate synthase	2.2.1.7	K01662	SmTc_5909
	DXS2	1-deoxy-D-xylulose-5-phosphate synthase	2.2.1.7	K01662	SmTc_7421
	DXS3	1-deoxy-D-xylulose-5-phosphate synthase	2.2.1.7	K01662	SmTc_10119
	DXS4	1-deoxy-D-xylulose-5-phosphate synthase	2.2.1.7	K01662	SmTc_13486
	DXR	1-deoxy-D-xylulose-5-phosphate reductoisomerase	1.1.1.267	K00099	SmTc_8142
	ISPD	2-C-methyl-D-erythritol 4-phosphate cytidylyltransferase	2.7.7.60	K00991	SmTc_23281
	ISPE	4-diphosphocytidyl-2-C-methyl-D-erythritol kinase	2.7.1.148	K00919	SmTc_19780
	ISPF	2-C-methyl-D-erythritol 2,4-cyclodiphosphate synthase	4.6.1.12	K01770	SmTc_11729
	GCPE	(E)-4-hydroxy-3-methylbut-2-enyl-diphosphate synthase	1.17.7.1	K03526	SmTc_845
	ISPH	4-hydroxy-3-methylbut-2-en-1-yl diphosphate reductase	1.17.7.4	K03527	SmTc_757
	IDI1	isopentenyl-diphosphate delta-isomerase	5.3.3.2	K01823	SmTc_744
	IDI2	isopentenyl-diphosphate delta-isomerase	5.3.3.2	K01823	SmTc_1157
	GPPS1	geranyl diphosphate synthase	2.5.1.1	K14066	SmTc_17467
	GPPS2	geranyl diphosphate synthase	2.5.1.1	K14066	SmTc_6570
Secoiridoid	GES	geranyl diphosphate diphosphatase	3.1.7.11	K20979	SmTc_70847
	G10H	geraniol 10-hydroxylase	1.14.13.152	K15099	SmTc_41455
	8HGO	8-hydroxygeraniol oxidoreductase	1.1.1.324		SmTc_81115
	IS	iridoid synthase	1.3.1.99	K20144	SmTc_35128
	IO	iridoid oxidase			SmTc_29298
	7-DLGT	7-deoxyloganetic acid glucosyl transferase	2.4.1.323		SmTc_15909
	DL7H	7-deoxyloganic acid hydroxylase			SmTc_8300
	LAMT	loganic acid O-methyltransferase	2.1.1.50		SmTc_23275
	SLS1	secologanin synthase	1.3.3.9	K13400	SmTc_4315
	SLS2	secologanin synthase	1.3.3.9	K13400	SmTc_16024
