# Supplementary material for: Deep sequencing and transcriptome analyses to identify genes involved in secoiridoid biosynthesis in the Tibetan medicinal plant Swertia mussotii
Source: Sci Rep. 2017 Feb 22;7:43108. doi: 10.1038/srep43108 (PMC5320516; doi:10.1038/srep43108)
Supplement: Supplementary Figure S1,S2,S3, Table S1,S2,S10 and S11 [file srep43108-s1.pdf]

Supplementary information

Original Research Article

Deep sequencing and transcriptome analyses to identify genes involved in secoiridoid biosynthesis in Tibetan medicinal plant *Swertia mussotii*

Yue Liu[1,2 §], Yi Wang[3 §], Fengxian Guo[1], Lin Zhan[1], Toni Mohr[4], Prisca Cheng[4], Naxin Huo[5], Ronghui Gu[1], Danning Pei[1], Lihong Chen[1], Li Tang[1], Chunlin Long[2] *, Luqi Huang[2] *, Yong Q. Gu[4]

[1]College of Life and Environmental Sciences, Minzu University of China, Beijing 100081, China

[2]National Resource Center for Chinese Materia Medica, China Academy of Traditional Chinese Medicine, Beijing 100700, China

[3]USDA-ARS, Plant Gene Expression Center, Albany, CA 94710, U.S.A.

[4]USDA-ARS, Western Regional Research Center, Albany, CA 94710, U.S.A.

[5]Department of Plant Science, University of California Davis, Davis, CA 95616, U.S.A.

[§]Contributed equally to the work

Corresponding authors
Chunlin Long
College of Life and Environmental Sciences
Minzu University of China
No. 27 South Zhongguancun Street, Haidian District
Beijing, P.R.China, 100081
Phone: (+8610)68932633
Fax:(+8610)68936927
E-mail: long@mail.kib.ac.cn

Luqi Huang
National Resource Center for Chinese Materia Medica
China Academy of Traditional Chinese Medicine
Beijing, P.R.China, 100700
Phone: (+8610) 64089801
Fax:(+8610) 64089801
E-mail:huangluqi01@126.com

Supplementary Figure S1. Alignment of HMGR amino acid sequences from *S. mussotii*.

Supplementary Figure S2. Alignment of SmSLS1 and SmSLS2 with other species. **a**, the alignment of SLS1 amino acid sequences between *Swertia mussotii* and *Nothapodytes nimmoniana*. **b**, the alignment of SLS2 amino acid sequences between *Swertia mussotii* and *Catharanthus roseus.*

Supplementary Figure S3. LC-MS profile of sweroside, swertiamarin and gentopicroside in different tissues of Swertia mussotii. Sweroside, m/z:359.1337, swertiamarin, m/z:375.1286, gentopicroside, m/z:357.1180. R, root; S, stem; L, leaf; F, flower.

Supplementary Table S1. Overview of *S. mussotii* transcriptome sequencing and de novo assemble results

Supplementary Table S2. Summary of functional annotation of *S. mussotii* transcripts by BLAST search against public databases.

Supplementary Table S3. Annotation of *S. mussotii* transcripts based on Blast to TrEMBL, Swiss-Prot and COG database.

Supplementary Table S4. *S. mussotii* transcript sequences corresponding to transcript factors.

Supplementary Table S5. Annotation of the assembled contigs GO and KEGG.

Supplementary Table S6. Gene function classification in KEGG database.

Supplementary Table S7. Identification of SSR motifs in the assembled sequences of *S. mussotii.*

Supplementary Table S8. Normalization of *S. mussotii* transcript reads using RPKM.

Supplementary Table S9. Enriched KEGG pathways for DETs in *S. mussotii* (P-value <0.05)

Supplementary Table S10. Expression and Sequence Comparison of Secoiridoid Biosynthesis Genes from S. mussotii and S. japonica

Supplementary Table S11. Primers used in Real-time PCR experiment.

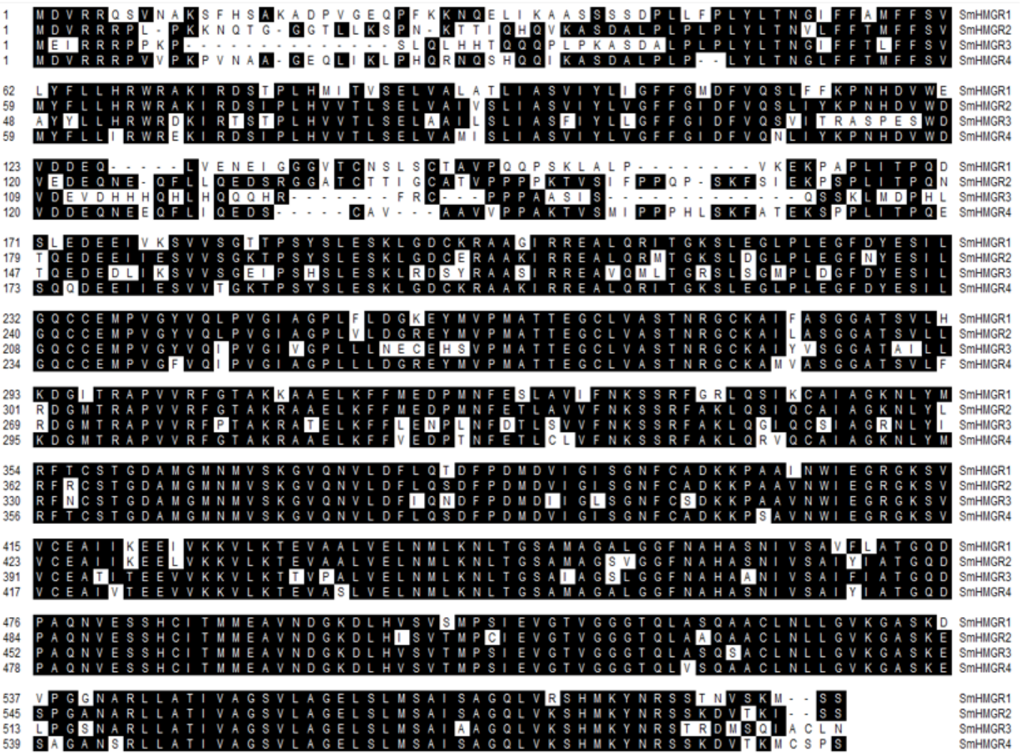

Supplementary Figure S1. Phylogeny and alignment of HMGR amino acid sequences from *S. mussotii*.

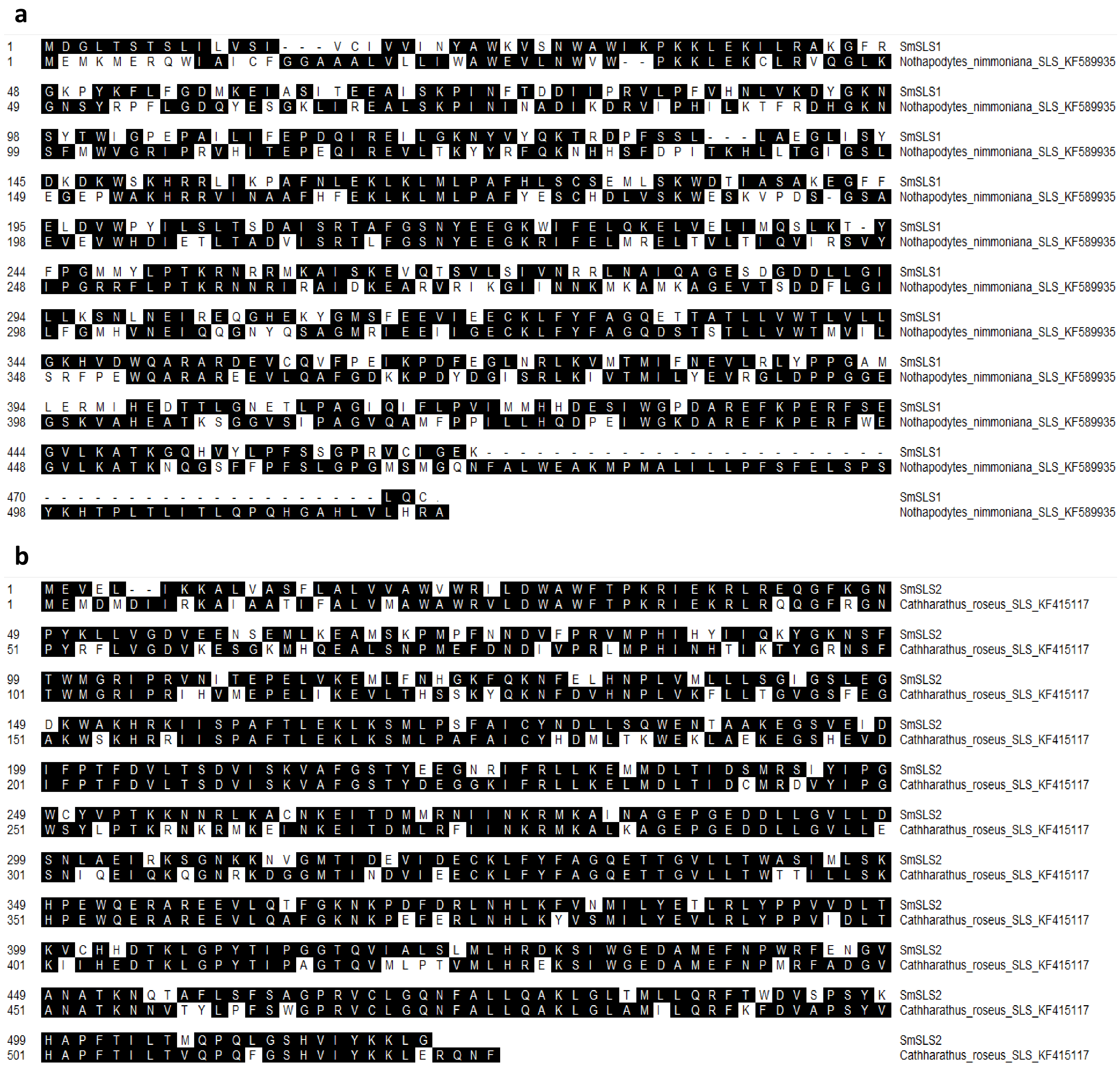

Figure S2. Alignment of SmSLS1 and SmSLS2 with other species. a, the alignment of SLS1 amino acid sequences between *Swertia mussotii* and *Nothapodytes nimmoniana*. b, the alignment of SLS2 amino acid sequences between *Swertia mussotii* and *Catharanthus roseus*.

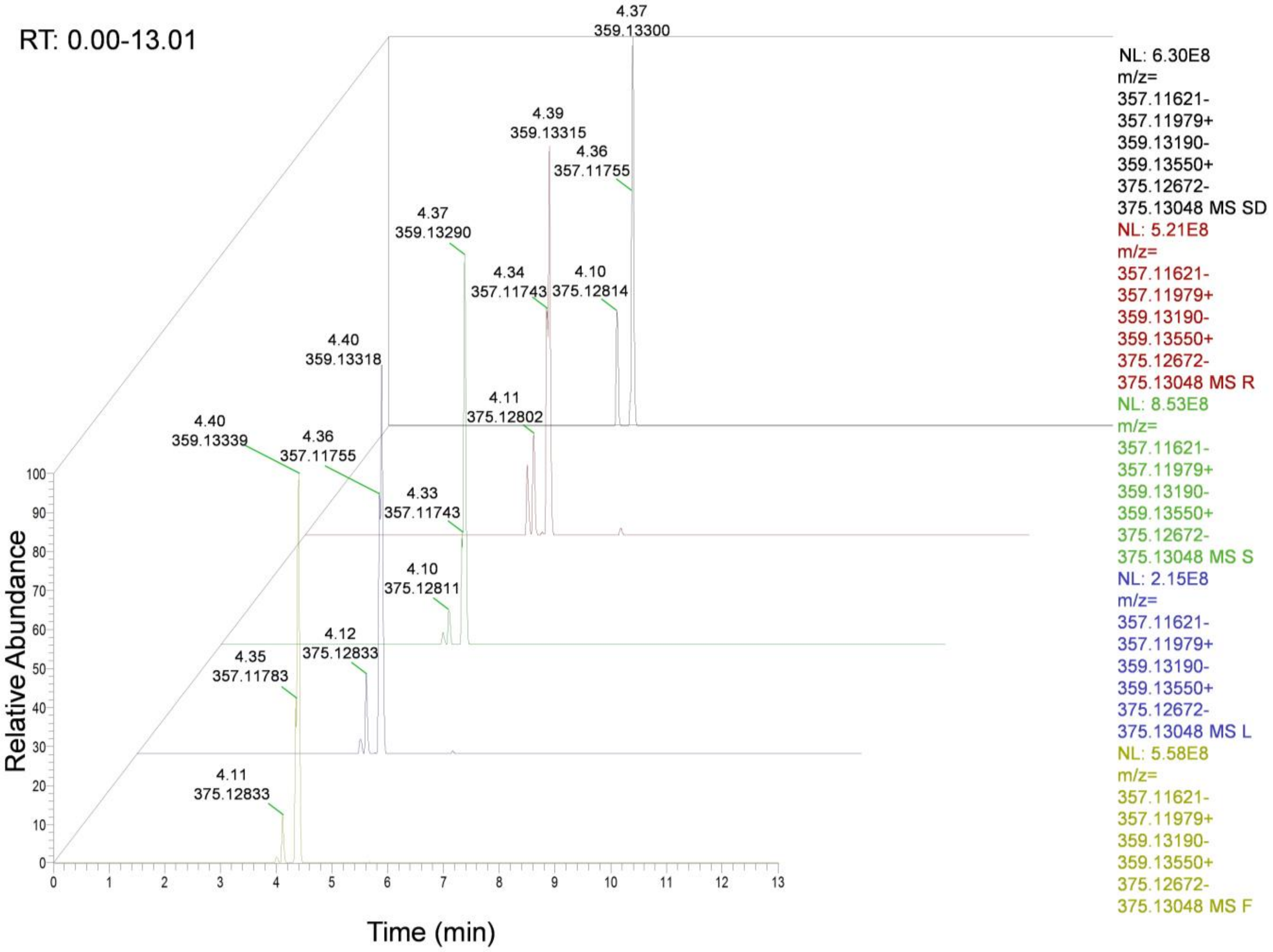

Supplementary Figure S3. LC-MS profile of sweroside, swertiamarin and gentopicroside in different tissues of *Swertia mussotii*. Sweroside, m/z:359.1337, swertiamarin, m/z:375.1286, gentopicroside, m/z:357.1180. SD, standard; R, root; S, stem; L, leaf; F, flower.

Supplementary Table S1. Overview of transcriptome sequencing and de novo assemble results

	**Root**	**Stem**	**Leaf**	**Flower**
Raw reads	262,348,938	379,017,528	369,525,968	284,489,346
Q30 percentage	92.22%	92.49%	91.63%	92.84%
Clean reads	241,934,818	350,544,954	338,593,514	264,133,962
Mapped reads	240,763,125 (91.77%)	338,906,256 (89.42%)	317,234,508 (85.85%)	250,941,234 (88.21%)
**After assembly**				
Total tanscripts	98,613			
Max length	13,875			
N50	1,085			
Percent GC	41.0%			
Total bases	81,753,344			

Supplementary Table S2. Summary of functional annotation of contigs from BLAST searches against public databases.

Database	Number of transcripts	Percentage
TrEMBL	30,489	30.92%
Swiss-Prot	11,593	11.76%
COG	11,175	11.33%
PlnTFDB	7,481	7.59%
GO	22,288	22.60%
KEGG	7,654	7.76%

Supplementary Talbe S10 Expression and sequence comparison of secoiridoid biosynthesis genes from *S. mussotii* and *S. japonica*

Gene	ID in S. mussotii	Expression ratio(leaf/root)	Pathway	ID in S. japonica	Expression ratio(leaf/root)	Pathway	Nucleotide Identity(%)
G10H	SmTc_41455	14.84	Secoiridoid	Unigene25762	9.99	Secoiridoid	97.32
IO	SmTc_29298	8		Unigene42532	26.08		97.68
IS	SmTc_35128	10.38		Unigene60394	13.26		97.58
7DLGT	SmTc_15909	0.14		Unigene39083	4.43		80.61
DL7H	SmTc_8300	2.09		Unigene7657	6.68		97.88
SLS2	SmTc_16024	5.4		Unigene62022	2.26		97.46
8HGO	SmTc_81115	12.27		Unigene45111	9.38		98.06
GES	SmTc_70847	20.03		Unigene54075	34.71		97.65
LAMT	SmTc_23275	0.65		Unigene40400	41.35		98.45
DXS1	SmTc_5909	1.25	MEP	Unigene59810	1.12	MEP	94.90
DXS2	SmTc_7421	0.73		Unigene47735	19.94		98.44
DXS3	SmTc_10119	0.71		Unigene59810	1.12		91.06
DXS4	SmTc_13486	0.97		Unigene11238	0.52		96.55
DXR	SmTc_8142	1.07		Unigene18958	3.06		96.53
ispD	SmTc_23281	0.96		Unigene39320	7.32		97.07
ispE	SmTc_19780	1.53		Unigene51912	2.8		97.44
ispF	SmTc_11729	1.52		Unigene41267	8.37		97.00
gcpE	SmTc_845	1.22		Unigene75010	3.67		97.98
ispH	SmTc_757	1.58		Unigene17496	6.17		97.98
IDI1	SmTc_744	1.01		Unigene38315	3.3		97.35
IDI2	SmTc_1157	0.82		Unigene38315	3.3		85.59
GPPS1	SmTc_6570	1.23		Unigene63348	1.39		97.50
GPPS2	SmTc_17467	0.89		Unigene63348	1.39		94.24
ACCT1	SmTc_2083	0.8	MVA	Unigene51665	1.11	MVA	96.85
ACCT2	SmTc_5029	0.81		Unigene59810	1.12		94.00
ACCT3	SmTc_33675	0.32		Unigene51665	1.11		90.47
HMGS1	SmTc_5929	0.75		Unigene11688	1.38		96.65
HMGS2	SmTc_7106	0.86		Unigene9398	4.3		96.67
HMGS3	SmTc_8664	0.78		Unigene17425	0.26		97.98
HMGR1	SmTc_1585	0.85		Unigene74519	0.9		97.23
HMGR2	SmTc_10138	0.23		Unigene40130	0.24		97.69
HMGR3	SmTc_15910	0.66		Unigene58422	1.31		97.27
HMGR4	SmTc_22303	1.01		Unigene10800	2.58		98.69
MVK	SmTc_7721	0.67		Unigene41645	0.47		97.17
MVD1	SmTc_2684	0.83		Unigene49879	0.52		98.04
MVD2	SmTc_2834	0.68		Unigene49880	1.18		92.62
PMK	SmTc_5121	0.86		Unigene46007	0.73		96.52

Supplementary Table S11 Primers used in all Real-Time PCR experiments

Gene name	Transcript No	Primer name	Sequence (5' to 3')	Product size /bp
G10H	SmTc_41455	G10H 999F	CGGAAAAGGAAAGGCGGTAG	102
		G10H 1100R	AAGAGTGGGACTGGTGGATG	
IS	SmTc_35128	IS 1F	GTCTCCAAACCGGTAGCAAA	199
		IS 1R	CCGAAAATCAGTGCAGGTCT	
IO	SmTc_29298	IO 1246F	AGGGATCCAGATGCTTGGAA	141
		IO 1386R	AGCCAATGGAAAACCGACAC	
7DLGT	SmTc_15909	7DLGT 106F	GTGCTTGTTCTTTCTGGGCT	130
		7DLGT 235R	CGACGCCTGAGGAAGTTTTC	
DL7H	SmTc_8300	7DLH 708F	GATGCGATGGAGTTCAACCC	146
		7DLH 853R	GCCATTGCCATCTTTGCTTC	
SLS1	SmTc_4315	SLS1 856F	GATGGGGATGACTTGCTTGG	200
		SLS1 1143R	CGAGCTTGCCAGTCTACATG	
SLS2	SmTc_16024	SLS 856F	GAGCCAGGCGAGGATGATTT	159
		SLS 1014R	CAAAACTCCGGTGGTTTCTT	
Tubulin	SmTc_275	Tubulin 784F	CCAACTCCAAGATGCCACTT	148
		Tubulin 931R	TCCTAGCACCAGAGGAAACC	
